# Of LAP, CUPS, and DRibbles – Unconventional Use of Autophagy Proteins for MHC Restricted Antigen Presentation

**DOI:** 10.3389/fimmu.2015.00200

**Published:** 2015-04-29

**Authors:** Christian Münz

**Affiliations:** ^1^Viral Immunobiology, Institute of Experimental Immunology, University of Zürich, Zurich, Switzerland

**Keywords:** Atg, exocytosis, endocytosis, unconventional secretion, TLRs

## Abstract

Macroautophagy delivers cytoplasmic constituents for lysosomal degradation. Because MHC class II molecules are loaded with lysosomal products for CD4^+^ T-cell stimulation, macroautophagy supports intracellular antigen processing onto MHC class II molecules. The molecular machinery of macroautophagy, however, does not only support this autophagic antigen processing, but seems to also modify extracellular antigen uptake for MHC class II presentation, antigen exocytosis, and packaging for improved cross-presentation onto MHC class I molecules. The different membrane trafficking pathways with LC3-associated phagocytosis, compartment for unconventional protein secretion, and DRibbles as well as the role that autophagic proteins play in them will be discussed in this review.

## Introduction

Autophagy describes several pathways, by which cytoplasmic constituents gain access to lysosomal degradation (1). During one of these autophagic pathways, called macroautophagy, a dedicated vesicle, the autophagosome, is formed, which engulfs with two membranes its cytoplasmic cargo and then fuses with lysosomes for degradation of its inner autophagosomal membrane and its contents.

## The Molecular Machinery of Macroautophagy

The extensive membrane remodeling events that first generate the cup-shaped isolation membrane, which then closes to autophagosomes and gets directed to fusion with late endosomes or lysosomes, require >30 essential proteins, which were termed autophagy-related gene (Atg) products. They are grouped in distinct complexes (ULK1 complex, Atg9L, the class III PI3K complex, the WIPI–Atg2 complex, the Atg12 conjugation complex, and the Atg8 conjugation complex). The Atg1/ULK1 complex (ULK1/2, Atg13, Atg101, and FIP200) is under metabolic control and can be activated by AMP activated protein kinase (AMPK) and inhibited by target of rapamycin (TOR), both through phosphorylation. The ULK1 complex induces macroautophagy during nutrient depletion to recycle cellular constituents for survival. This constitutes the canonical function of macroautophagy. The ULK1 complex in turn phosphorylates members of the class III PI3K complex (Vps34, Atg14L, Beclin-1, and Vps15), including Atg6/Beclin-1, which activates phosphatidylinositol 3-phosphate (PI3P) deposition at membrane sites like the ER, where autophagosomes are generated. Atg9L most likely recruits membranes to these sites after phosphorylation by the ULK1 complex. The PI3P marks recruit WIPI–Atg2 complexes to these membrane sites, which function as landing platforms for the Atg12–Atg5/Atg16L1 complexes via direct binding of Atg16L1 to WIPI2. These interactions lead to autophagosome initiation.

For autophagosome elongation, the Atg12–Atg5/Atg16L1 complexes assemble after ubiquitin-like coupling of Atg12–Atg5 via the E1- and E2-like enzymes, Atg7 and Atg10, and binding of Atg16L1 to the resulting conjugate. The Atg12–Atg5/Atg16L1 complex fulfills the E3-like enzymatic activity for the other ubiquitin-like system that couples Atg8 to phosphatidylethanolamine (PE) in the assembling autophagosome membrane called the phagophore or isolation membrane. Prior to conjugation five amino acids are removed from the C-terminus of Atg8 by the protease Atg4, and Atg8 is then activated and conjugated by Atg7 and Atg3. Atg8 has eight mammalian orthologs (two splice variants of LC3A, LC3B, LC3C, GABARAP, and GABARAPL1-3). The PE coupled forms of these Atg8 orthologs can be distinguished from their cytosolic form by SDS gel electrophoresis. LC3B-PE is assessed most often for this purpose and migrates faster (LC3B-II) than its cytosolic form (LC3B-I). These autophagic protein tags are conjugated to the outer and inner membrane of the developing autophagosome and seem to mediate membrane fusion events during phagophore elongation and recruit substrates into autophagosomes via LC3 interacting regions (LIRs). Once the autophagosome is completed the Atg12–Atg5/Atg16L1 complex dissociates and Atg4 also cleaves Atg8 from the outer membrane. These molecular events lead to autophagosome completion.

For autophagosome maturation, however, residual Atg8 at the outer autophagosome membrane might facilitate migration along microtubules and fusion with lysosomes via recruitment of FYVE and coiled coil domain containing 1 (FYCO1) (2) and pleckstrin homology domain containing protein family member 1 (PLEKHM1) proteins (3). Alternatively, FYCO1 and PLEKHM1 could also be recruited by the lipid composition of the outer autophagosomal membrane, including PI3P. PLEKHM1 in turn seems to recruit the homotypic fusion and vacuole protein sorting (HOPS) complex, the GTPase Rab7 that is involved in late endosome and autophagosome fusion with lysosomes, and even the SNARE syntaxin 17 (STX17), which executes outer autophagosome membrane fusion with lysosomes. Autophagosome fusion with lysosomes is again regulated by type III PI3K complexes containing Vps34, Beclin-1, and VPS15. PI3K complexes containing Rubicon inhibit, whereas UVRAG containing complexes accelerate autophagosome fusion with lysosomes. These findings indicate that some components of the fusion machinery are also recruited by PI3P to the autophagosomal membrane. Thus, macroautophagy utilizes a sophisticated membrane remodeling and fusion machinery, which recently has been found to support other membrane trafficking events. This involvement of Atg proteins in endocytosis and exocytosis for antigen processing toward MHC restricted antigen presentation will be discussed in this review.

## LC3-Associated Phagocytosis

**Figure 1:**
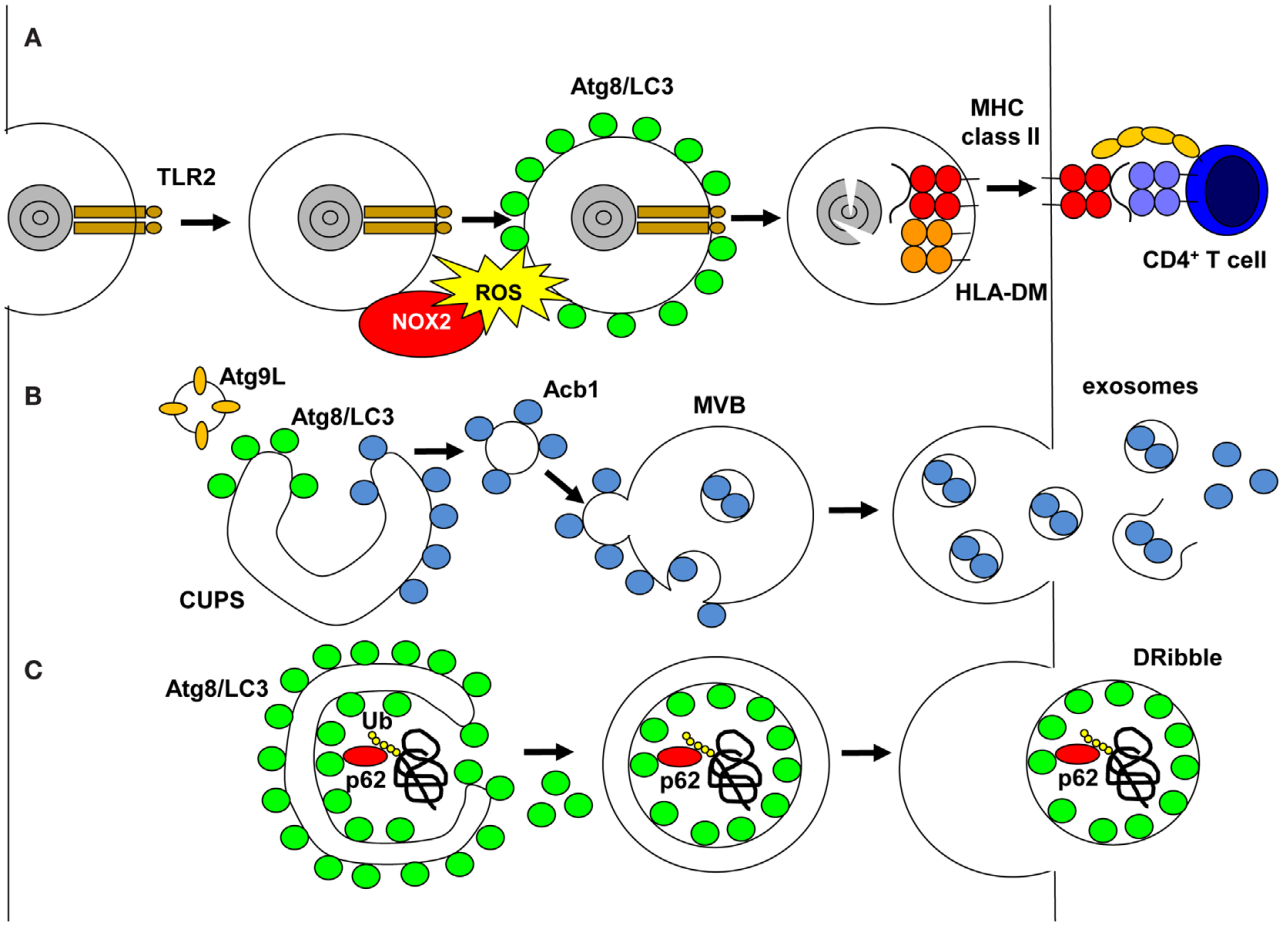
**Unconventional pathways that use autophagic proteins**. **(A)** LC3-associated phagocytosis (LAP) is engaged on TLR2 ligand phagocytosis, which recruits NADPH oxidase (NOX2). NOX2 produce reactive oxygen species (ROS), which are required to recruit or maintain Atg8/LC3 at the phagosomal membrane. Atg8/LC3 needs to be cleaved from the phagosomal membrane for phagosomes to fuse with MHC class II loading compartments, and their cargo is degraded by lysosomal hydrolysis and fragments loaded onto MHC class II molecules for stimulation of CD4^+^ T-cells. **(B)** During unconventional secretion of proteins without signal peptides for co-translational insertion into the ER, Atg8/LC3 and Atg9L cooperate to expand the compartment for unconventional protein secretion (CUPS). Proteins to be secreted, like acyl-CoA binding protein 1 (Acb1), bind on the cytosolic side to these membranes, then vesicles from the CUPS fuse with multivesicular bodies (MVBs). Acb1 is internalized into intravesicular membranes by invagination and then released in exosomes after MVB fusion with the cell membrane. **(C)** Proteasomal inhibition diverts ubiquitinated proteins, including defective ribosomal products, into autophagosomes via sequestosome 1/p62 binding to polyubiquitin and Atg8/LC3. The inner autophagosomal membrane with this cargo is released as defective ribosomal products-containing autophagosome-rich blebs (DRibbles) if lysosomal degradation is inhibited.

MHC class I and II molecules present peptide products of proteasomal and lysosomal proteolysis to CD8^+^ cytotoxic and CD4^+^ helper T-cells, respectively (4). Classically, endocytosed material is delivered to lysosomes and its fragments are then presented on MHC class II molecules. One prominent endocytic pathway is phagocytosis and Atg proteins have been found to regulate it during LC3-associated phagocytosis (LAP) (5) (Figure 1). During LAP, its cargo needs to engage a subset of phagocytic and/or pathogen-associated molecular pattern (PAMP) receptors to recruit NADPH oxidase 2 (NOX2) to the phagosomal membrane (6, 7). These receptors can be members of the toll-like receptor (TLR) family, primarily TLR2, or the C-type lectin Dectin-1, the T-cell immunoglobulin mucin protein 4 (TIM4) or Fc receptors for immunoglobulins (5, 7–10). NOX2 either allows coupling of LC3 to phagosomes or prevents its uncoupling by Atg4, and this LC3 lipidation is independent of the Atg1/ULK1 complex (8). Irrespective of the exact mechanism, LC3 is coupled to the cytosolic side of the limiting phagosomal membrane (7) and influences the fate of these LAP phagosomes. In some cell types, primarily mouse macrophages, LAP phagosomes seem to fuse more rapidly than LC3 negative phagosomes with lysosomes (5, 10, 11), whereas in human macrophages, conventional and plasmacytoid dendritic cells (DCs), LAP vesicles seem to be stabilized and can fuse with TLR containing endosomes (7, 12). Acceleration of LAP phagosome fusion with lysosomes might be because of more efficient transport along microtubules via LC3 binding to FYVE and coiled-coil domain containing 1 (FYCO1) protein (11). Stabilized LAP phagosomes do not recruit Rab7 and do not seem to contain classical autophagy cargo like the LIR-positive sequestosome 1/p62 protein (7). Prior to delayed fusion of LAP vesicles with lysosomes, LC3 is uncoupled from the phagosomal membrane (7). This delayed phagosomal processing might be beneficial for continuous MHC class II antigen presentation of phagocytosed antigens. Indeed, LAP formation was required for prolonged antigen processing of Candida antigens for MHC class II presentation to specific human CD4^+^ T-cell clones *in vitro* (7). Similarly, Dectin-1 bound antigens were also more efficiently presented to mouse CD4^+^ T-cells (9). Finally, CD4^+^ T-cell priming after antigen injection into mice with Atg5-deficient DCs was compromised as compared to wild-type mice, suggesting that LAP improves MHC class II presentation of exogenous antigen *in vivo*, in addition to the role of Atg5 in endogenous antigen processing for MHC class II presentation (13). Indeed, retention of phagocytosed antigens by LAP might account in part for more efficient antigen presentation after attenuated lysosomal degradation (14). Therefore, regulation of phagocytosis by autophagic proteins seems to enhance MHC class II antigen presentation of extracellular antigens.

## Compartment for Unconventional Protein Secretion

Autophagy-related gene proteins are also involved in unconventional membrane trafficking in the opposite direction of phagocytosis, namely signal peptide independent secretion (15). A number of proteins leave eukaryotes not via the classical ER to Golgi pathway and lack signal peptides for co-translational insertion into the ER. These include acyl-CoA binding protein 1 (Acb1), interleukin-1β, interleukin-33, tissue transglutaminase, galectin-3, macrophage migration inhibitory factor, and insulin-degrading enzyme (15). Recently, it was found that Acb1 and IL-1β require autophagic proteins for their secretion, namely Atg1, 5, 6, 7, 8, 9, 11, 12, and 17 (16–18). Moreover, release of microbial peptides and secretory lysosomes was also found to be dependent on this autophagic core machinery (19–21). Interestingly, the secretion of Acb1 during yeast and ameba starvation requires the formation of a membrane structure close to the ER exit site that seems to be composed of cis- and trans-Golgi membranes and is called the compartment for unconventional protein secretion (CUPS) (22, 23) (Figure 1). It contains PI3P, Atg9, Atg8, the Golgi-associated protein GRASP65 and Vps23 of the ESCRT-1 complex for multivesicular body (MVB) formation. Moreover, after starvation the CUPS is absorbed into the ER (23). Therefore, the CUPS might be a continuous membrane, which is elongated like the isolation membrane with the help of Atg proteins, but might never form a double-membrane surrounded autophagosome. Instead, the cargo for unconventional secretion seems to get attached to the cytosolic side of the membrane (15). Small Acb1-coated vesicles might then get released from CUPS and fuse with an endosomal compartment, on the surface of which they require the ESCRT-1 complex for invagination of the endosomal membrane to generate MVBs. These MVBs fuse then with the cell membrane to release exosomes that contain the unconventionally secreted substrate. Indeed, the surface membrane SNARE Sso1 has been found to be required for unconventional Acb1 secretion (17). Moreover, part of the IL-1β released from activated macrophages is membrane engulfed and therefore protease resistant (24). This unconventional secretion pathway could also give rise to immunostimulatory exosomes, which have been proposed for efficient antigen delivery vesicles that already contain antigenic peptide loaded MHC molecules (25).

## Defective Ribosomal Products-Containing Autophagosome-Rich Blebs

Such exosomal fractions might be further modified by autophagosomes, which are not degraded in lysosomes. Indeed, loss of Atg5, Atg6/Beclin-1, or Atg12 compromised tumor and influenza A virus-infected cells to donate antigens for efficient cross-presentation (26, 27). Ubiquitinated proteins and defective ribosomal products, including viral and tumor antigens, were enriched in vesicular fractions of cell supernatants after blocking proteasomal and lysosomal degradation (28) (Figure 1). In the presence of proteasomal inhibition, ubiquitinated substrates of the proteasome seem to aggregate and get incorporated into autophagosomes via sequestosome1/p62, a protein that anchors ubiquitinated proteins to LC3 for autophagic substrate recruitment (29). Blocking lysosomal degradation might then generate autophagolysosomes with MVB characteristics, from which the inner autophagosomal membranes and their cargo might be released into the vesicular supernatant fraction. Similar pathways of arrested macroautophagy might be used by viruses to exit cells (30–33). These vesicular fractions were coined defective ribosomal products-containing autophagosome-rich blebs (DRibbles) and can be endocytosed in a CLEC9A-dependent fashion by antigen presenting cells (28). DRibbles seem to be more efficient for cross-presentation on MHC class I molecules to CD8^+^ T-cells than cell lysates and soluble antigens (34). DRibbles were similarly processed for MHC class II presentation to CD4^+^ T-cells like whole cell lysates, but better than soluble viral antigens. The more efficient MHC class I cross-presentation could be further enhanced in human peripheral blood mononuclear cells by GM-CSF and TLR3 agonist addition (34). Furthermore, DRibbles immunization of mice protected from challenge with melanoma, lung carcinoma, breast carcinoma, and sarcoma cell lines (29, 35). Thus, frustrated macroautophagy of proteasomal substrates might lead to secretion of DRibbles, which could be a superior antigen format for cross-presentation on MHC class I molecules.

## Conclusion

LAP, CUPS, and DRibbles bear witness to non-canonical functions of Atg proteins. They have in common with macroautophagy that they regulate membrane trafficking and cargo delivery to topologically extracellular compartments (endosomes or cell supernatants during endocytosis and exocytosis, respectively). Atg proteins might facilitate membrane fusion events and recruit substrates and adaptor proteins during the trafficking of LAP- and CUPS-derived vesicles as well as during DRibbles secretion. A better understanding of these non-canonical Atg pathways should provide insights into how groups of Atg proteins can be used by different cellular pathways in a modular fashion to achieve vesicle elongation, fusion with other vesicles and substrate recruitment. This knowledge would then also provide a better understanding of macroautophagy itself, the pathway that defined Atgs. Because MHC-restricted antigen presentation relies on cellular trafficking events to produce antigenic fragments, load them into MHC peptide binding grooves, and then transport the products to the cell surface for T-cell recognition, interference with the various Atg-assisted pathways undoubtedly modulates T-cell recognition of antigens and these pathways should be harnessed during vaccination.

## Conflict of Interest Statement

The author declares that the research was conducted in the absence of any commercial or financial relationships that could be construed as a potential conflict of interest.
